# Experimental infection of indigenous North African goats with goatpox virus

**DOI:** 10.1186/s13028-021-00574-2

**Published:** 2021-03-04

**Authors:** Jihane Hamdi, Zahra Bamouh, Mohammed Jazouli, Meryem Alhyane, Najet Safini, Khalid Omari Tadlaoui, Ouafaa Fassi Fihri, Mehdi El Harrak

**Affiliations:** 1 Department of Research and Development, Multi-Chemical Industry Santé Animale, Lot. 157, Z I, Sud-Ouest (ERAC) B.P.: 278, 28810 Mohammedia, Morocco; 2 Department of Microbiology, Immunology and Contagious Diseases, Agronomic and Veterinary Institute Hassan II, Madinat Al Irfane, 6202 Rabat, Morocco

**Keywords:** Clinical scoring, Experimental infection, Goatpox, Pathogenicity

## Abstract

**Background:**

Goatpox is a viral disease caused by infection with goatpox virus (GTPV) of the genus *Capripoxvirus*, *Poxviridae* family. Capripoxviruses cause serious disease to livestock and contribute to huge economic losses. Goatpox and sheeppox are endemic to Africa, particularly north of the Equator, the Middle East and many parts of Asia. GTPV and sheeppox virus are considered host-specific; however, both strains can cause clinical disease in either goats or sheep with more severe disease in the homologous species and mild or sub-clinical infection in the other. Goatpox has never been reported in Morocco, Algeria or Tunisia despite the huge population of goats living in proximity with sheep in those countries. To evaluate the susceptibility and pathogenicity of indigenous North African goats to GTPV infection, we experimentally inoculated eight locally bred goats with a virulent Vietnamese isolate of GTPV. Two uninfected goats were kept as controls. Clinical examination was carried out daily and blood was sampled for virology and for investigating the antibody response. After necropsy, tissues were collected and assessed for viral DNA using real-time PCR.

**Results:**

Following the experimental infection, all inoculated goats displayed clinical signs characteristic of goatpox including varying degrees of hyperthermia, loss of appetite, inactivity and cutaneous lesions. The infection severely affected three of the infected animals while moderate to mild disease was noticed in the remaining goats. A high antibody response was developed. High viral DNA loads were detected in skin crusts and nodules, and subcutaneous tissue at the injection site with cycle threshold (Ct) values ranging from 14.6 to 22.9, while lower viral loads were found in liver and lung (Ct = 35.7 and 35.1). The results confirmed subcutaneous tropism of the virus.

**Conclusion:**

Clinical signs of goatpox were reproduced in indigenous North African goats and confirmed a high susceptibility of the North African goat breed to GTPV infection. A clinical scoring system is proposed that can be applied in GTPV vaccine efficacy studies.

## Background

Goatpox is a viral disease of small ruminants caused by infection with goatpox virus (GTPV) of the genus *Capripoxvirus*, a member of the family *Poxviridae* [1]. Capripoxviruses have a linear, double stranded DNA genome, approximately 150 kilo base pairs (bp) in length, and encode for 156 genes. Members of this group, which include sheeppox virus (SPPV) and lumpy skin disease virus (LSDV), are genetically closely related, sharing 96% of nucleotide homology among isolates [2].

Infection with GTPV and SPPV are of major concern in goats and sheep. When the disease spreads into a susceptible population, the viruses remain restricted to one host species. However, SPPV or GTPV can transmit between sheep and goats in mixed flocks, causing severe clinical signs in one species and less severe disease in the other [3–5].

Goatpox cause economic losses through mortality, decline in milk yield, damage to hides and trade restrictions [6, 7]. Transmission of GTPV is direct between animals, through respiratory aerosols due to close contact between infected and naïve animals. GTPV may also spread by contamination of abrasions and other skin lesion [3], and experiments have shown that mechanical transmission by flies may occur [8, 9].

After an incubation period of 5 to 14 days, GTPV infected goats develop fever, loss of appetite, depression, ocular and nasal discharge and “pox lesions”. The cutaneous pox lesions develop on the head, mouth, fore and hind limbs, and over hairless parts of the body. They progress through macular, papular, vesicular and pustular stages until scabs are formed. Papules can develop in the mucous membranes. Lesions are mainly observed in the skin, oronasal surfaces, gastro-intestinal tract and lungs, but can also be seen in conjunctiva, vulva, prepuce, scrotum and udder [10–12]. Papules in conjunctiva and nasal cavity may ulcerate and be accompanied by a mucopurulent discharge. The most severely affected animals may develop pneumonia and mastitis and pregnant females may abort. Secondary bacterial infections are also common and death may occur at any stage of the disease [10].

Alike infection with SPPV, morbidity and mortality rates due to GTPV infection depend on the breed, virus strain and immunity. Mild infections are common among indigenous breeds in endemic areas, but the morbidity can reach 70–90% with a mortality ranging from 5 to 10%. Severe disease with morbidity and mortality of up to 100% may occur in young, stressed and naïve animals [13]. The virus is highly stable, persisting in the environment for up to six months [14].

Goatpox and sheeppox occur in Africa north of the Equator, the Middle East and many parts of Asia, including Afghanistan, Kyrgyzstan, Pakistan, Kazakhstan, India, Nepal and parts of China, and the Russian Federation [15, 16]. In North Africa, goats represent 18% of the small ruminant population, i.e., around 12 million goats [17]. Despite the large population of goats and clinical surveillance programs established in North Africa, no cases of goatpox have been identified [18–21]. This may be due to the absence of circulating virulent GTPV strains in the region, natural resistance of the indigenous goat breeds or absence of transmission of SPPV from sheep to goats.

In this study, we experimentally inoculated indigenous North African goats with a virulent isolate of GTPV to (1) assess the susceptibility of local goat breeds to virulent GTPV, (2) develop a clinical scoring system and (3) examine the antibody response and viral load.

## Methods

GTPV inoculation of the goats was carried out in accordance with international guidelines for care and handling of experimental animals, chapter 7.8 of the Terrestrial Animal Health Code and Directive 2010/63/UE of the European Commission [22, 23]. The protocol was approved by the Internal Ethic Committee for Animal Experiment at Multi-Chemical Industry Santé Animale (2018-MCI-013).

### Viral isolate

The virulent isolate of GTPV was provided by the Pirbright Laboratory (Vietnamese strain, isolated from lung tissue from a naturally GTPV infected goat in 2004). The virus was passed three times on chorio-allantoic membranes of fertilized hen’s eggs according to Munyanduki et al. [24], followed by three passages on primary testis cells, maintained in Dulbecco’s Modified Eagle’s Medium with 10% irradiated fetal calf serum [25]. The viral suspension was harvested after five days of incubation at 35 °C. The inoculum had a titre of 10^5.4^ 50% tissue culture infective doses (TCID _50_) per mL.

### Animals

Six months old locally bred North African male goats (n = 10), of around 30 kg body weight, that were raised specifically for experimentation, were housed in an animal biological safety level 3 containment facility in Morocco, were fed a complete balanced diet and had access to water ad libitum. The goats were divided into two groups (8 inoculated goats and 2 negative controls), and housed in separate isolation boxes. The goats were prescreened and found to be negative for GTPV specific antibodies by a virus neutralization test (VNT) as explained below. The goats were kept for 5 days for acclimatization before starting the experiment.

### Study design

Eight goats were inoculated subcutaneously in the right side of the neck with 0.5 mL of GTPV inoculum. Two control animals were injected subcutaneously with 0.5 mL of phosphate buffered saline (PBS) solution as placebo. The goats were observed daily for clinical signs and skin lesions until euthanasia. Rectal temperatures were taken daily until 14 days post-infection (pi). Semi-quantitative clinical scoring was done to evaluate the clinical expression and development of skin lesions. The clinical scoring system ranged from 0 to 2, 3 or 5 based on the severity of behavioral changes, rectal temperature, injection site alteration, feed intake, size and distribution of cutaneous lesions and presence and amount/character of oculo-nasal discharge (Table 1). A total cumulative clinical score was made per animal each day, with a possible maximum daily score of 23. Humane end-points were defined before starting the experiment. To avoid suffering animals were euthanized following appearance and persistence for 24 h of absence of feeding/watering and inactivity or aggravation of lesions (humane end-points). Animals were euthanized by anesthesia through intravenous application of xylazine and intramuscular injection of ketamine followed by exsanguination. Animals not reaching the humane end-points were euthanized at the end of the study (day 18 pi) in a similar way.

**Table 1:** Scoring of clinical signs and skin lesions after experimental subcutaneous goatpox virus inoculation

Clinical signs	Score
General behavior	
Normal	0
Stillness	1
Get up when approached	2
Recumbent	3
Feed intake	
Normal	0
Reduce appetite	1
Anorexia	2
Anorexia and not drinking	3
Nodule at injection site	
Absent	0
Small (1–2 cm)	1
Medium (2–3 cm)	2
Large (> 3 cm)	3
Temperature (℃)	
< 39.5	0
39.5 ≤ T < 40.0	1
40.0 ≤ T < 40.5	2
40.5 ≤ T < 41.0	3
T ≥ 41.0	4
Cutaneous lesions	
Absent	0
Single nodule < 5 mm	1
Single nodule > 10 mm	2
Small nodules in head/flank	3
Generalized nodules < 10 mm	4
Generalized nodules ≥ 10 mm	5
Oculo-nasal discharge	
Normal	0
Mild and watery	1
Serous and profuse	2
Muco-purulent	3
Salivation	
Normal	0
Moderate	1
Profuse	2

For serology, blood samples were collected in plain vacuum tubes via jugular venipuncture using an 18-G needle at day 7 and 14 pi. Tubes were then placed in vertical position at room temperature for 1 h. After clotting, blood was centrifuged at 1500 rounds per minute (RPM) for 15 min, serum was collected, aliquoted and stored at -20 °C until analysis. Whole blood samples were collected in EDTA tubes every third day until day 12 pi and kept at 4 °C until analysis.

### Serology

Sera were analyzed by VNT as described in the OIE Terrestrial Manual [26]. Sera were heat- inactivated at 56 °C for 30 min and serial 1:3 dilutions of serum were mixed with a constant dose of GTPV (2.5 log_10_) and incubated for 1 h at 37 °C. The serial dilutions were then inoculated onto lamb testis cell suspensions and incubated for 7 days at 37 °C with 5% of CO_2_, for observation of neutralization of cytopathic effect (CPE). The neutralizing antibody titer was calculated in accordance with the Reed and Muench method [27], which determines 50% end-point by serial dilution using the following formula: log_10_ 50% end point dilution = log_10_ of dilution showing 50% of infected cell cultures—(difference of logarithms × logarithm of dilution factor) [27].

### Necropsy

The goats were necropsied and tissue specimens were sampled for virology. These consisted of subcutaneous tissue at the injection site (primary nodule), cutaneous lesions not related to injection site (secondary nodules in the skin), cutaneous crusts overlaying secondary nodules, trachea, lung, heart, liver, spleen, testis, rumen, abomasum, reticulum, omasum, duodenum, kidney, pulmonary and mesenteric lymph nodes.

### Virology

Collected tissues were minced using sterile scissors and grounded after adding 2 volumes of PBS with 5% penicillin and streptomycin. The suspension was then centrifuged at 3500 RPM for 30 min. The supernatant was aliquoted and stored at − 80 °C for real-time polymerase chain reaction (RT-PCR) and virus isolation.

Viral DNA was extracted from the tissue samples and blood using ISOLATE II Genomic DNA Kit (Bioline, London, UK). Samples were evaluated using a quantitative polymerase chain reaction (qPCR) TaqMan assay that amplified and detected 89 bp region from open reading frame 074 which encodes the intracellular mature virion envelope protein P32 within SPPV, GTPV and LSDV [2, 28]. Real-time PCR was conducted with the TaqMan Universal PCR Master Mix Kit (Applied Biosystems, Foster City, CA, USA). The reaction was performed in 96-well optical reaction plates, containing: 12.5 µL of 2 × TaqMan Universal PCR Master Mix, Real-time PCR buffer, 1 µL of each primer Forward 5′-AAA ACG GTA TAT GGA ATA GAG TTG GAA-3′ and Reverse 5′-AAA TGA AAC CAA TGG ATG GGA TA-3′ (10 µM), 0.5 µL of probe FAM-TGG CTC ATA GAT TTC CT-MGB (10 µM) [29], 5 µL of template (DNA) and 5 µL nuclease free water.

The qPCR assay was run in ABI7500 (Applied Biosystems) with the following cycling conditions: 95 °C for 10 min, followed by 40 cycles of PCR at 95 °C for 15 s and 60 °C for 1 min.

The viral inoculum was validated by inoculation of viral dilutions on lamb testis cells and observation of CPE after 7 days of incubation at 37 °C in at atmosphere containing 5% CO_2_.

Viral isolation was also attempted from subcutaneous tissue of one of the affected goats (no 335), using 80% confluent lamb testis cells permissive to capripoxviruses [30].

## Results

### Disease progression—clinical signs and skin lesions

During the acclimation period, the goats remained clinically normal with rectal temperatures from 38.5 to 39.5 °C. Within three days after inoculation, all infected goats (8/8) developed a primary nodule, i.e., a 2–3 cm diameter subcutaneous swelling at the injection site.

**Fig. 1:**
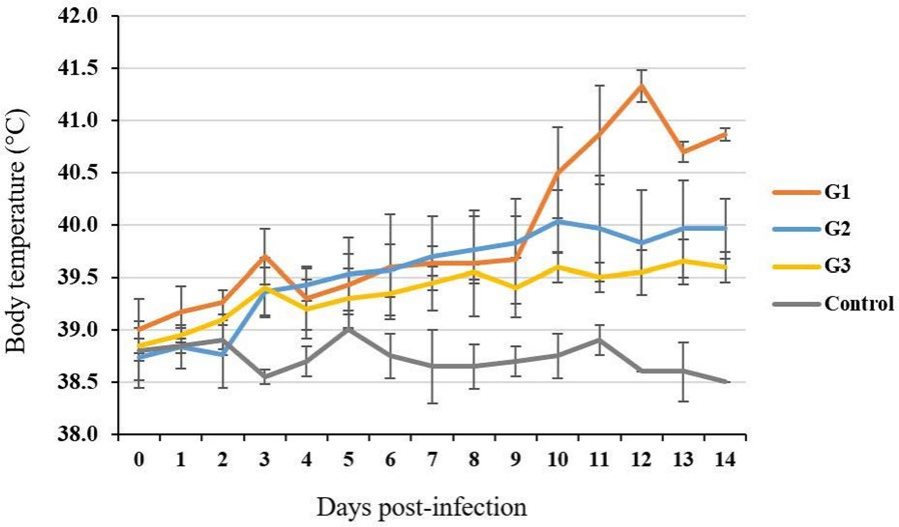
Average daily rectal temperature of goats inoculated with a Vietnamese goatpox virus (GTPV) isolate (n = 8) and controls (n = 2). The inoculated goats were divided into three groups (G) according to their clinical score. G1 represents goats with highest clinical scores (n = 3); G2 represents goats with moderate scores (n = 3), and G3 represents goats with lowest scores (n = 2)

**Fig. 2:**
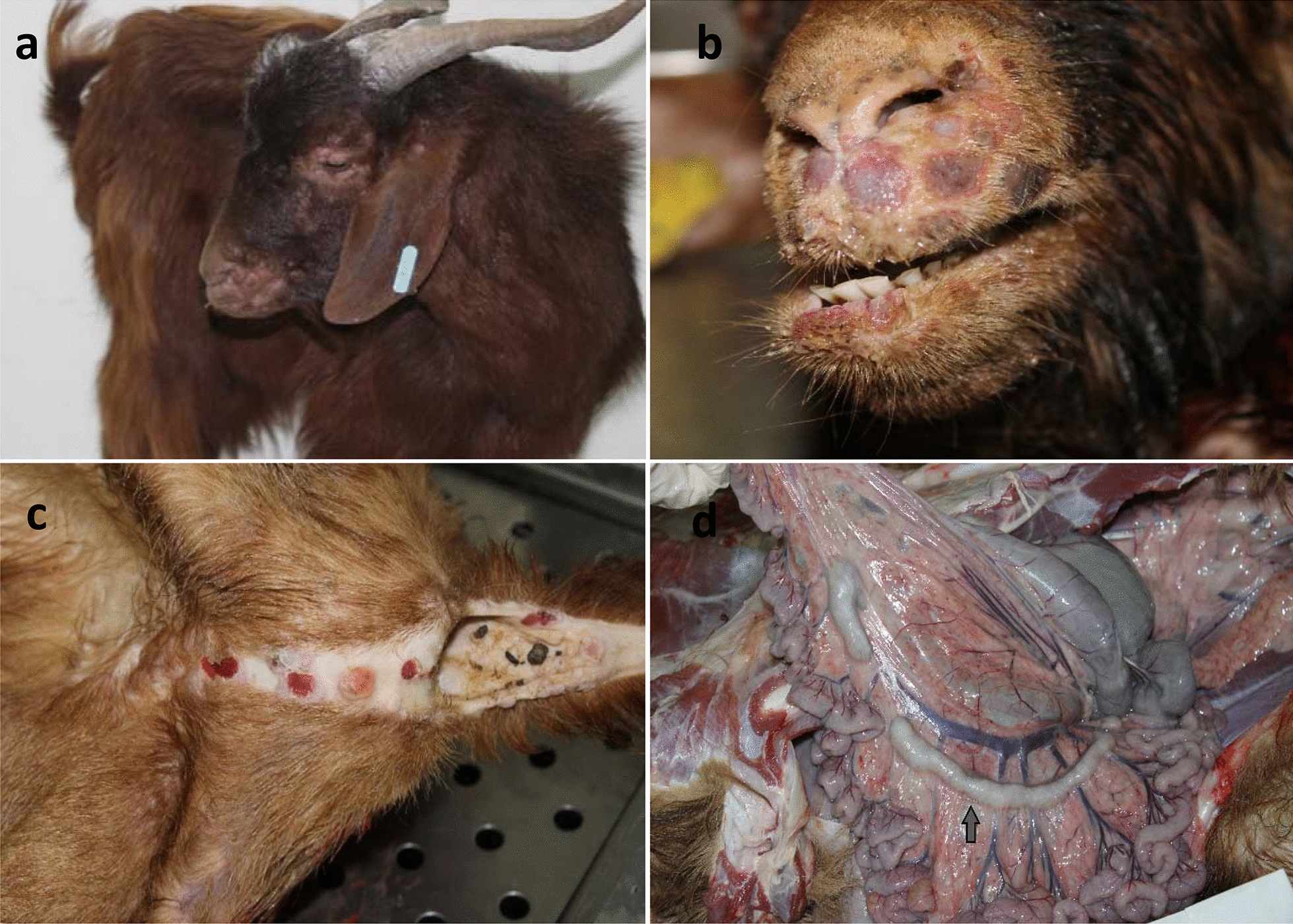
Lesions following experimental infection of goats with a Vietnamese goatpox virus isolate. **a**, **b** Goats developed papules and nodules in the skin of head and ears. **c** Pox lesion with haemorrhagic papules on the ventral part of the tail and perineum. **d** Small intestine and mesentery with enlargement of mesenteric lymph nodes (arrow) and congestion of blood vessels

Three goats (129, 335 and 373) developed hyperthermia from day 3 pi and the rectal temperature peaked at day 12 pi (average 41.3 °C) (Fig. 1). Focal skin nodules on eyelids, around the mouth and nose were observed by day 8 pi and generalized to the flanks, ears, neck and hairless areas by day 10 pi (Fig. 2a–c). The nodules evolved into vesicles and crusts by day 14 pi. Bilateral oculo-nasal discharge with crusting around nares appeared by day 10 pi, followed by salivation at day 14 pi. Starting from day 7 pi, the goats showed decreased appetite and varying degrees of reduced activity. Once the animals developed clinical signs, they were monitored twice a day by veterinarians who ensured their feeding and watering. At day 15 pi, the three goats developed anorexia and became depressed; they were consequently euthanized within 24 h, i.e., at day 16 pi as improvement was not observed. At this stage, each goat had a total clinical scoring of 22 (Table 2).

**Table 2:** Experimental days with highest clinical scoring after inoculation of goats with goatpox virus (n = 8) and controls (n = 2)

Goat no.	Group 1^a^			Group 2^a^			Group 3^a^		Control	
	129	335	373	269	287	261	752	265	759	267
Score										
Post-infection day of highest score	16	16	16	18	18	18	18	18	12	17
Behavior	3	3	3	2	2	1	1	1	1	0
Feed-intake	2	2	2	1	0	0	0	0	0	0
Nodule at injection site	3	3	3	3	3	3	2	2	0	0
Temperature	4	4	4	3	1	2	1	1	0	0
Cutaneous lesions	5	5	5	5	5	5	3	3	0	0
Oculo-nasal discharge	3	3	3	2	2	2	1	1	0	1
Salivation	2	2	2	0	0	0	0	0	0	0
Total clinical scoring	22	22	22	16	13	13	8	8	1	1

^a^Group 1, Group 2, Group 3: Inoculated goats were retrospectively divided into 3 groups, based on severity of clinical signs at the end of the experiment

In three other goats (261, 284 and 269), temperatures between 39.3 and 40.5 °C were recorded between day 6 and day 14 pi (Fig. 1). At day 10 pi they developed extensive secondary skin nodules that were restricted to the head and the flank regions, i.e. less extensive than in goats 129, 335 and 373. They were euthanized at the end of the study at day 18 pi; at this stage they had reached clinical scores of 13–16 (Table 2).

In the remaining inoculated goats (752 and 265), mild temperature increases were registered from day 7 pi (39.5 °C). The following days, temperatures varied between 39.4 and 39.7 °C (Fig. 1). Small skin nodules appeared on the head and hairless areas at day 12 pi. The animals were euthanized at day 18 pi with a clinical score of 8 (Table 2). The uninfected control animals (759 and 267) remained healthy during the whole experiment.

Based on the severity of the clinical signs, infected goats were divided into 3 groups (G1-3),

G1: goats with severe clinical disease (animals 129, 335 and 373), G2: goats with moderate signs (animals 261, 284 and 269) and G3: goats with mild disease (animals 752 and 265).

**Fig. 3:**
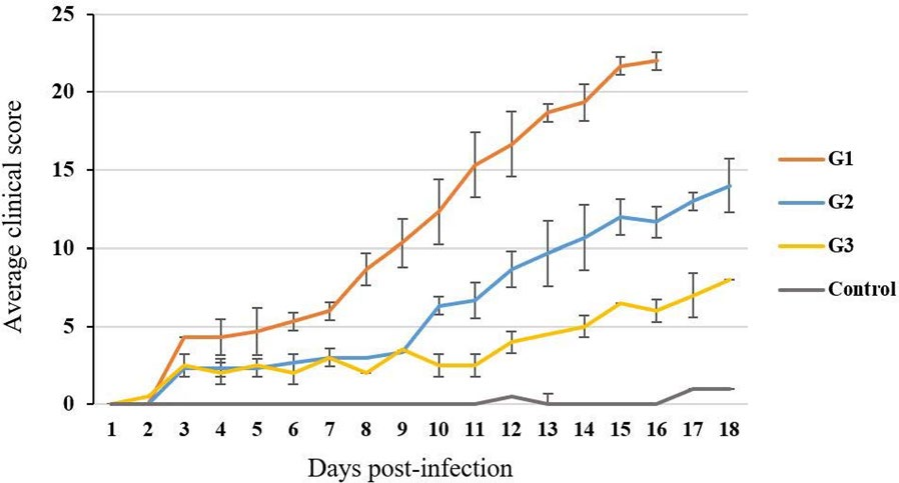
Progression of clinical score (average) per day for goats inoculated with a Vietnamese goatpox virus (GTPV) strain (n = 8) and controls (n = 2). The inoculated goats were divided into three groups (G) according to their clinical score. G1 represents goats with highest clinical scores (n = 3); G2 represents goats with moderate scores (n = 3), and G3 represents goats with lowest scores (n = 2)

The average clinical score per day was calculated for each group, showing a clear difference between the 3 inoculated groups regarding disease progression (Fig. 3). Goats from G1 showed the most severe disease and their clinical score reached 22. In the 2 other groups, clinical scores of individual animals did not exceed 16.

### Gross pathology

The most severely affected goats (G1) showed prescapular and inguinal lymph node enlargement. At the injection site, the subcutaneous tissue appeared congested and edematous. Nodules and petechiae were observed in the skin of the head and flank. The mucous membranes of nares, mouth and eyelids were ulcerated. In goats of G1 and G2, the mesenteric lymph nodes were enlarged. The greater omentum was congested and had petechiae in goat 129 of G1 (Fig. 2d). The lungs did not show any gross pathology in any animal.

### Virology and serology

Viral genome quantification results (expressed by cycle threshold (Ct)) are shown in Table 3. The highest DNA content was found in skin crusts (Ct 14.6), subcutaneous tissue at the injection site (Ct 19.4), testis (Ct 19.1), secondary nodules (Ct 20.8) and trachea (Ct 22.9) in goats from G1. PCR analysis showed a lower viral load in the lung (Ct 35.7) and liver (Ct 35.1). There was no apparent difference in viral load between the groups. Blood tested negative for GTPV at all times.

**Table 3:** PCR results in cycle threshold (Ct) values of tissue samples from goats inoculated with a Vietnamese goatpox virus isolate (n = 8) and controls (n = 2)

Goat No.	Group 1^a^			Group 2^a^			Group 3^a^		Control	
	335	129	373	269	287	261	265	752	759	267
Tissue	Ct value									
Subcutaneous tissue at injection site	19.4	18.2	18.9	20.2	20.5	NS	NS	NS	NS	NS
Skin crusts	16.6	16.6	16.5	18.3	14.9	14.6	17.1	17.3	NS	NS
Secondary nodules	NS	NS	NS	20.8	22.1	22.6	24.7	31.4	NS	NS
Testis	NS	19.1	NS	31.4	NS	NS	NS	NS	NS	NS
Trachea	22.9	25.6	NS	23.5	32.8	30.8	36.4	36.3	–	–
Lung	–	–	–	36.2	–	–	35.7	–	–	–
Pulmonary lymph node	29.9	–	36.2	33.7	–	–	31.8	–	–	–
Heart	–	–	34.9	34.1	NS	NS	NS	NS	–	–
Spleen	–	–	NS	32.1	34.4	–	–	–	–	–
Liver	–	–	NS	–	–	–	35.1	–	–	–
Rumen	NS	NS	NS	29.8	NS	NS	NS	NS	NS	NS
Abomasum	37.2	–	NS	35.1	NS	NS	NS	NS	NS	NS
Reticulum	30.8	NS	NS	29.3	NS	NS	NS	NS	NS	NS
Omasum	24.3	35.9	NS	29.8	NS	NS	NS	NS	NS	NS
Duodenum	–	–	NS	33.9	–	NS	NS	NS	NS	NS
Mesenteric lymph node	34.4	–	NS	NS	34.1	36.5	36.7	34.7	–	–
Kidney	34.7	–	NS	–	–	–	–	–	–	–
Blood	–	–	–	–	–	–	–	–	–	–

Tissues were sampled at day 16 (group 1) post infection (pi) or day 18 pi (group 2, group 3, Control)

*NS* not sampled

–: Negative, i.e. below detection level

^a^Group 1, Group 2, Group 3: Inoculated goats were retrospectively divided into 3 groups, based on severity of clinical signs at the end of the experiment

VNT analyses of serum showed presence of antibodies on day 14 pi in all inoculated goats, with titers higher than 1.5 DN_50_ (equivalent dilution 1/30) in 7/8 animals. The remaining goat (129 from G1) showed a weak response of 1.26 DN_50_ (equivalent dilution 1/18). None of the control goats seroconverted.

**Fig. 4:**
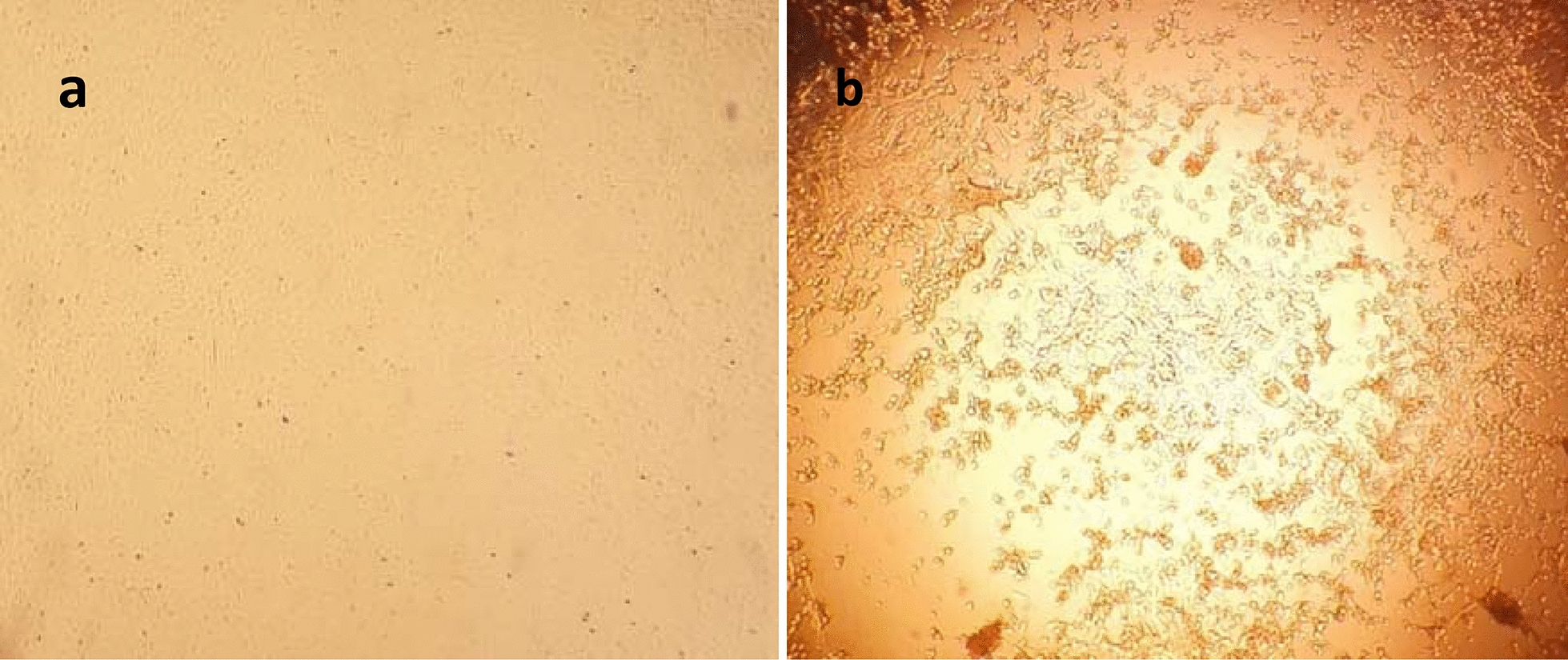
Cells infected with goatpox virus. **a** Non-infected primary testis cells. **b** Primary testis cells infected with goatpox virus isolate showing cytopathic effect consisting of clumping and rounding of cells in lysis plaques

GTPV was also detected by virus isolation on lamb testis cells from the subcutaneous tissue of a severely infected goat (335). Cytopathic effect appeared at day 4 pi and reached 80% at day 6 pi, consisting of clumping and rounding of cells in lysis plaques (Fig. 4).

## Discussion

This is the first study on indigenous North African goat breed’s susceptibility to a virulent strain of GTPV. Three inoculated goats (G1) developed a severe disease, which lead to their euthanasia before the end of the experiment, while other goats displayed moderate to mild clinical signs despite being of the same age and breed and inoculated with the same strain and dose. These results are similar to what was observed by Babiuk et al. [12] using the same virus strain, as severe clinical signs were observed in 3 of 9 experimentally infected Boer cross-bred goats. It seems that the GTPV strain causes a disease with varying degree of severity [29, 31], which has also been observed for the related LSDV [32, 33]. This may be explained by phylogenetic similarities between GTPV and LSDV [34, 35]. From our study, we can conclude that indigenous North African goats are as susceptible as other breeds if exposed to a virulent strain of GTPV.

The used clinical scoring represents a tool to quantify each stage of the disease in goats and can be used in challenge study for vaccine potency testing or to compare strain virulence and breed susceptibility. Clinical scorings have been also applied for bluetongue [36], African swine fever [37], peste des petits ruminants [38, 39] and lumpy skin disease [40]. The scoring allows us to compare the difference in clinical expression of the disease in goats, which could be attributed to individual susceptibility of the animals.

The clinical signs were similar to what was observed in other experimental GTPV infections of goats, and at necropsy, skin and mucosa of the oral cavity, nares and eyelids were affected, as also reported by Babiuk et al. [12] and Bowden et al. [29]. However, lesions in parenchymal organs such as spleen, liver and lungs were found in these studies, which was not the case in our experiment. Using PCR, high level of viral DNA was detected in the skin from where the virus was isolated, confirming subcutaneous tropism of GTPV [12, 29].

## Conclusion

This study reproduced clinical goatpox in an indigenous North African goat breed using a virulent Vietnamese GTPV isolate and confirmed a susceptibility of this breed to GTPV. As indigenous goats are susceptible to GTPV, there is a high risk of spread of goatpox in North Africa if a virulent strain is introduced. Surveillance for goatpox should therefore be regularly conducted. A clinical scoring system was proposed to grade the severity of disease.

## Data Availability

The datasets used during the current study are available from the corresponding author on reasonable request.

## Abbreviations

CaPV: Capripoxvirus
CPE: CytoPathic effect
Ct: Cycle threshold
DN_50_: Dose neutralizing 50
GTPV: Goatpox virus
LSDV: Lumpy skin disease virus
OIE: World Organization for Animal Health
PBS: Phosphate buffered saline
pi: Post-infection
RPM: Rounds per minute
RT-qPCR: Real-time reverse transcriptase-polymerase chain reaction
qPCR: Quantitative polymerase chain reaction
SPPV: Sheeppox virus
TCID_50_: Tissue culture infective dose
VNT: Virus neutralization test
